# Emerging Role of Extracellular Vesicles in the Pathophysiology of Multiple Sclerosis

**DOI:** 10.3390/ijms21197336

**Published:** 2020-10-04

**Authors:** Ettore Dolcetti, Antonio Bruno, Livia Guadalupi, Francesca Romana Rizzo, Alessandra Musella, Antonietta Gentile, Francesca De Vito, Silvia Caioli, Silvia Bullitta, Diego Fresegna, Valentina Vanni, Sara Balletta, Krizia Sanna, Fabio Buttari, Mario Stampanoni Bassi, Diego Centonze, Georgia Mandolesi

**Affiliations:** 1 Synaptic Immunopathology Lab, Department of Systems Medicine, Tor Vergata University, 00133 Rome, Italy; ettoredolcetti@hotmail.it (E.D.); brunoa.neuro@gmail.com (A.B.); livia.guadalupi@gmail.com (L.G.); f.rizzo@med.uniroma2.it (F.R.R.); Silvia.Bullitta@uniroma2.it (S.B.); valentina_vanni@hotmail.it (V.V.); balletta.sara@gmail.com (S.B.); krizia.sanna@live.it (K.S.); 2 Synaptic Immunopathology Lab, IRCCS San Raffaele Pisana, 00163 Rome, Italy; msllsn00@uniroma2.it (A.M.); antonellag79@gmail.com (A.G.); diego.fresegna@gmail.com (D.F.); georgia.mandolesi@uniroma5.it (G.M.); 3 Department of Human Sciences and Quality of Life Promotion, University of Rome San Raffaele, 00163 Rome, Italy; 4 Unit of Neurology, IRCCS Neuromed, Pozzilli (Is), 86077 Pozzilli, Italy; f.devito.molbio@gmail.com (F.D.V.); silviacaioli@yahoo.it (S.C.); fabio.buttari@gmail.com (F.B.); m.stampanonibassi@gmail.com (M.S.B.)

**Keywords:** extracellular vesicles, exosome, microvesicles, multiple sclerosis, experimental autoimmune encephalomyelitis, neuroinflammation, multiple sclerosis therapy

## Abstract

Extracellular vesicles (EVs) represent a new reality for many physiological and pathological functions as an alternative mode of intercellular communication. This is due to their capacity to interact with distant recipient cells, usually involving delivery of the EVs contents into the target cells. Intensive investigation has targeted the role of EVs in different pathological conditions, including multiple sclerosis (MS). MS is a chronic inflammatory and neurodegenerative disease of the nervous system, one of the main causes of neurological disability in young adults. The fine interplay between the immune and nervous systems is profoundly altered in this disease, and EVs seems to have a relevant impact on MS pathogenesis. Here, we provide an overview of both clinical and preclinical studies showing that EVs released from blood–brain barrier (BBB) endothelial cells, platelets, leukocytes, myeloid cells, astrocytes, and oligodendrocytes are involved in the pathogenesis of MS and of its rodent model experimental autoimmune encephalomyelitis (EAE). Most of the information points to an impact of EVs on BBB damage, on spreading pro-inflammatory signals, and altering neuronal functions, but EVs reparative function of brain damage deserves attention. Finally, we will describe recent advances about EVs as potential therapeutic targets and tools for therapeutic intervention in MS.

## 1. Introduction

Extracellular vesicles (EVs) are an heterogeneous group of membrane-bound vesicles released from most body cells into the extracellular space, playing important roles in intercellular communication, both locally and systemically [1,2]. Mediators expressed on the surface of the EVs or transported in their lumen are responsible of the intercellular communication and can change depending on the type of stimuli received. The transporting cargo includes protein, lipids, and RNAs across long distances in the whole body [3,4]. The current research has tried to classify EVs based on their size and cargo. It has been recognized that EVs can contain over 40,000 different kind of proteins, nearly one-quarter of the known human proteome [5], including cytoplasmic enzymes, cytoskeleton molecules, adhesion molecules, signal transduction proteins, membrane trafficking molecules, heat-shock proteins, cytokines, chemokines, proteinases, and cell-specific antigens (Ags). Moreover, EVs contain messenger RNA (mRNAs), non-coding RNA (ncRNAs) including miRNAs, and even extra-chromosomal DNA [6]. Importantly, the EVs’ content largely reflects that of the parent cell, but it is still technically difficult to classify EVs on the basis of their cargo [7], establish their origin, differentiate them in subpopulation, or perform an individual particle analysis once isolated from biological fluids [4,8,9]. Those limitations have driven current research to classify EVs according to their dimension and shedding mechanisms. In particular, exosomes (diameter ranges from 50 nm to 150 nm) are stored within multivesicular endosomes (MVEs) as intraluminal vesicles and released in the extracellular space through the fusion of MVEs with the plasma membrane [4]. Their outward budding mechanism is predominantly mediated by the endosomal sorting complex required for transport (ESCRT) machinery or other “non-ESCRT dependent” machinery including sphingomyelinase (SMase) and tetraspanins [10]. Microvesicles (MVs) (diameter ranges from 50 nm to 1 µm), also known as “shedding microvesicles” or ectosomes, are generated through the direct outward budding of the plasmatic membrane and subsequent fission of plasma membrane blebs [4]. Their outward mechanism is still not completely understood and includes proteins such as SMase, calpain, scramblase, protein kinase C, and part of the ESCRT machinery [4]. Finally, a peculiar and relatively poorly known member of the EVs family is represented by the apoptosome (500 nm–2µm)—vesicular apoptotic bodies released following the disassembly of an apoptotic cell into subcellular fragments [11]. Throughout the text we will refer to EVs when both exosome and MVs are considered or when no better difference is mentioned in the recounted paper. On the contrariwise, whenever possible we will indicate whether the article deals specifically with exosome, MVs, or apoptosome.

The discovery of EVs as a new entity of intercellular communication has revolutionized the view of the regulatory properties of the immune and nervous systems that together with the endocrine signaling represent the primary regulators of distant tissue. EVs released from both immune and non-immune cells cover an important role in immune regulation. For example, studies showed that EVs derived from dendritic cells, B lymphocytes, and endothelial cells activate and stimulate T cells by antigen presentation, representing thus a promoter of adaptive immune response [12,13]. Similarly, regulatory T cells (Tregs) are capable to release exosomes providing another mechanism by which these cells can exert their immunosuppressive function. Due to their impact on immune system, EVs have been involved in inflammatory, autoimmune, and infectious disease pathology [12]. EVs have a relevant role also in the physiology of the nervous system mediating both local and long-distance intracellular cross-talk, especially the neuro-glia communication. Notably, during an intense firing, neurons release a large amount of EVs at the synaptic cleft to induce in astrocytes the upregulation of the excitatory amino acid transporter 2 (EAAT2; also known as GLT1) in order to remove excess of glutamate from the cleft [14,15]. Notably, EVs are also released by microglia, the resident immune cells of the CNS [1,10] that, in the presence of an increased extracellular concentrations of ATP, a molecule released from damaged cells, activate an inflammatory response [16]. Considering that neurons, glia, and peripheral immune cells form an integrative network to actively regulate immunological processes that affect brain functions, it is not surprising that EVs are involved in the pathophysiology of many diseases, including neurovascular, neurodegenerative, neuroinfectious, neurooncological, and psychiatric diseases [1,17,18,19]. Among those, multiple sclerosis (MS) represents a paradigmatic disease in which communication between the immune system and CNS is strongly compromised. EVs are emerging as important mediators of both pathological and reparative mechanisms in MS [20,21], and their bidirectional trafficking from the CNS in extra-CNS biological fluids, facilitated by blood-brain barrier (BBB) leaks associated to MS pathophysiology [22,23], prompted several investigators at consider EVs as potential diagnostic and prognostic biomarkers of this neurological disease [23].

In the present review, we provide an overview of the recent advances made in understanding the role of EVs in the different pathological mechanisms that characterize MS disease, considering both preclinical and clinical studies. Then, we focus on new intriguing potential therapeutic application of EVs in MS.

## 2. Multiple Sclerosis: Clinical Features and Pathophysiology

MS is a chronic inflammatory disease of the central nervous system (CNS), characterized by a wide variety of neurological symptoms including muscle weakness, sensory, visual and cerebellar deficits, cognitive impairments, and psychic symptoms such as fatigue and depression [24]. The clinical course of MS is classified by McDonald’s diagnostic criteria in two different phenotypes: relapsing–remitting and progressive [24]. The so-called relapsing remitting MS (RRMS) is the most common phenotype, and it is characterized by acute episodes of neurological deficits (relapse) followed by a return to baseline function (remission) of clinical symptoms. Over a long term follow-up, 15–30% of RRMS patients develops progressive disability, and this phenotype is classified as secondary progressive multiple sclerosis (SPMS). About 15% of MS patients directly develops a progressive phenotype from the outset and are classified as primary progressive multiple sclerosis (PPMS) patients [25]. Clinical classification also provides clinically isolated syndrome (CIS), a single clinical event compatible with MS that could both evolve in a RRMS or remain isolated, and radiologically isolated syndrome (RIS), an incidental finding of radiological signs of disease in the absence of clear clinical activity [26].

The etiology of MS is still unknown, but it is evident that a complex interaction between environmental, genetic, and epigenetic factors triggers an autoimmune reaction against the CNS compartment [24,27]. The most accredited hypothesis is that peripheral T and B lymphocytes, primed against a still-unknown antigen, drive a cross-reaction against CNS epitopes including oligodendrocytes proteins, such as myelin basic protein, proteolipid protein, and myelin oligodendrocyte glycoprotein (MOG). Animal modeling, despite several limitations, has been crucial to understand MS pathogenesis. In particular, the experimental autoimmune encephalomyelitis (EAE) has greatly contributed to our understanding of autoimmunity and of inflammation-induced neurodegenerative processes [28]. A hallmark of MS pathophysiology is a progressive BBB dysfunction that causes infiltration in the CNS of peripheral pathogenic T and B cells, antibodies, monocytes, and inflammatory mediators. The chain of inflammatory reaction triggered by infiltrating leucocytes leads to demyelination, axonal damage, and synaptic loss and dysfunction, named synaptopathy, ultimately resulting in a prominent neurodegeneration [27,29,30]. Moreover, pro-inflammatory mediators, including interferon-γ (INF-γ), interleukin-1β (IL-1β), and particularly, tumor necrosis factor-α (TNF-α), released by peripheral leucocytes and activated microglia and astrocytes, favorite adhesion of activated leucocytes on the endothelium by overexpression of nitric oxide and adhesion molecules (VCAM-1, E-selectin, and CD31/PECAM-1) [31]. Such inflammatory milieu leads to endothelial release of metalloprotease and proteolytic enzymes that contribute to BBB disruption, further increasing the trafficking of autoreactive T and B cells, antibodies, monocytes, and inflammatory mediators from vessels into the CNS. Radiological evidence of a leaky BBB is provided by the contrast (gadolinium; gad+) enhancement of active plaques in magnetic resonance imaging (MRI) [32]. White matter neuroinflammatory foci are indeed easily detectable during the acquisition of MRI, appearing as hyper-intense areas called “plaques” or demyelinating lesions. The demyelination process is partially counteracted by proliferation and migration of oligodendroglial precursor cells (OPCs, NG2+) to the lesion site, where they differentiate in mature oligodendrocytes (OLGs) and form the new myelin sheath [30]. However, in MS patients OPCs are often detained at the plaque edge, or they may differentiate into malfunctioning premyelinating OLGs [33]. The interplay between OPCs and glial cells seems to cover a crucial role in the remyelination process. In particular, a pro-regenerative microenvironment can be produced by a complex interaction between mesenchymal stem cells (MSC), microglia, astrocytes, and IL-4-releasing macrophages [34,35]. The presence of MSCs during neuroinflammation influences the release of IL-4, a cytokine involved into remyelinating processes, and favorites the expression of pro-regenerative genes by activated microglia [36]. On the other hand, in the absence of MSCs, primary microglia express elevated levels of pro-inflammatory genes and mediators, such as IL-1α, C1q, IL-1β, inducible nitric oxide synthase (iNOS) [36]. The establishment of a chronic inflammatory state, caused by a microenvironment enriched with activated microglia releasing pro-inflammatory cytokines, such as TNF-α [36], gradually impairs remyelinating processes as a result of altered OPC activation and recruitment to demyelinating lesions [35]. This process leads to a consistent axonal loss and is associated to clinical impairment and the progression of disease.

The MS brain is also affected by another degenerative but potentially reversible phenomenon, namely inflammatory synaptopathy. Clinical research using transcranial magnetic stimulation (TMS) [37], pre-clinical studies conducted on an EAE model [29,38], and more recently chimeric ex-vivo models [29,39] have highlighted the presence of an inflammation-dependent decrease in the gamma-aminobutyric acid (GABA) ergic tone and an increase in the glutamatergic transmission in several MS/EAE brain areas. Such a synaptic transmission unbalances results in a diffuse synaptic dysfunction and loss that is mediated by pro-inflammatory molecules released by peripheral immune system cells, microglia, and astroglia [29,37]. On the other hand, synaptic plasticity events may intervene as a compensatory mechanism to overcome synaptic damage, becoming exhausted in the MS progressive forms. The consequence of a long-lasting synaptic imbalance is an excitotoxic damage that triggers neurodegenerative processes [29,37].

## 3. Extracellular Vesicles as Potential Biomarkers in MS

Accumulating evidence highlights serum/plasmatic and CSF EVs as potential biomarkers of MS disease stages and of response to treatment (Table 1).

### 3.1. Serum and Plasma

In the attempt to identify blood-derived EVs as disease stage biomarkers, important associations emerged between EVs, mostly derived from platelet-(CD61+), leukocyte- (CD45+), monocyte (CD14+) [49], and endothelium-(CD51+ and CD31/PECAM-1+) cells [40] and disease activity, progression, and drug response. One of the first studies, conducted by Minagar in 2001, showed that plasma levels of CD31/PECAM-1+ EVs released by endothelial cells were significantly higher in RRMS patients during the active phase of disease compared to non-active RRMS patients and healthy control (HC) subjects. A positive correlation with active (gad+) lesions in MRI [40] emerged, suggesting that high levels of EVs detectable in the patients’ plasma may anticipate a radiological relapse of disease [40]. These results are in line with the observation that the CD31/PECAM-1 adhesion molecule is strictly involved in transendothelial migration of leukocytes during the inflammatory processes. Conversely, endothelial EVs CD51+ (integrin alpha-V) remained elevated during both exacerbation and remission, thus appearing to be a marker of chronic damage more than endothelial disruption [40]. Other clinical studies showed that endothelial EVs were significantly increased in the serum of both SPMS and RRMS patients, with higher levels in relapsing–remitting than progressive forms, thus reflecting a status of active peripheral inflammation rather than chronic neurodegeneration [42,45,50]. Interestingly, elevated plasma levels of conjugates between endothelial EVs and monocytes directly correlated with MRI gad+ lesions in a court of RRMS patients [41]. In vitro studies suggested that the binding of endothelial EVs to monocytes might promote their activation by enhancing monocytes’ migration through an endothelial monolayer [41]. The association between active MRI lesions and plasma levels of EVs was further supported by Jimenez and colleagues that observed an increase in endothelial EVs in MS patients during the clinical relapse phase compared to remission [44], suggesting endothelial EVs as potential biomarkers of BBB damage. Besides EVs derived from endothelial cells, Sheremata and colleagues, pointed out to an aberrant activation of platelets in MS as a secondary effect of a chronic endothelial damage showing a high count of CD41+ platelet EVs in MS plasma compared to HC [46]. These data were supported by the observation that RRMS in remission present higher levels of platelet EVs plasma levels, together with monocyte- and leukocyte-derived EVs, in comparison to SPMS and HC [45]

Previous studies in MS have assessed the content of circulating EVs and have demonstrated significant alterations in miRNA profiles and relationships to disease course [51,52,53] (Table 2). Ebrahimkhani and colleagues demonstrated that serum—exosome cargo of microRNA, with no specification of its cellular origin—was different in RRMS (miR-15b-5p, miR-451a, miR-30b-5p, miR-342-3p) and progressive MS patient sera (miR-127-3p, miR-370-3p, miR-409-3p, miR-432-5p) in comparison to HC [51]. Selmaj and colleagues observed a significant reduction in several serum-exosomal miRNAs (hsa-miR-122-5p, hsa-miR-196b-5p, hsa-miR-301a-3p, and hsa-miR-532-5p) during relapse in RRMS [53]. These miRNAs were also decreased in patients with a gadolinium enhancement in brain MRI. The authors also assessed in vitro the secretion of these miRNAs by peripheral blood mononuclear cells (PBMC) and observed a significant impairment in RRMS. Specific miRNA have also been implicated in potentially pathogenic effects on the immune system in MS [54,55] and therapeutic response [56].

One previous study evaluated the protein cargo of circulating EVs and noted alterations in myelin associated proteins [53]. Plasmatic and CSF PBMC-derived exosomal content of MOG directly correlated with radiological relapse of disease in RRMS and SPMS patients. The authors suggested that this peripheral release of EVs containing myelin antigen may sustain inflammation, peripheral activation, and migration of immune cells against CNS oligodendrocytes perpetuating the anti-myelin immune reactions [53].

### 3.2. Cerebrospinal Fluid

Due to its proximity to the CNS, the most direct source of biomarkers is CSF [59,60]. Biochemical and molecular analysis of the CSF has been indeed compared to a liquid biopsy of CNS, but lumbar puncture to collect this precious biological fluid is an invasive procedure not applicable for the follow-up of MS patients [61]. Furthermore, the relative low amount of EVs as well as the small volume of CSF available have made EVs isolation from CSF very challenging. However, researchers are developing new techniques to make CSF analysis a routine part of optimal MS clinical management and to find alternative and less invasive approaches [62]. In this regard, a recent work has compared the levels of microvesicles in CSF and in tears, supporting the latter as intriguing possible samples for the study of EVs [63].

Among the data obtained in the CSF, early observations by Scolding and colleagues revealed the presence of vesicles in the CSF of MS patients [64]. More recently, Verderio et al. confirmed these data by showing an overproduction of EVs in CSF in subjects with a diagnosis of CIS or MS versus HC [16]. MVs display neuronal, astrocytic, oligodendroglial, or microglia/macrophage markers, thus indicating that they originate from all these brain cells. By focusing on MVs of myeloid origin the authors found a positive correlation between macrophage/microglia EVs levels and radiological activity of MS disease. Notably, they showed a high degree of sensitivity and specificity in the distinction between CIS and HC and between active and non-active patients [16], suggesting macrophagic/microglial EVs as optimal biomarkers of neuroinflammation.

An important change in the number of CSF EVs and in their surface marker expression during active phases of MS was confirmed in a recent study, in particular a CSF EV’s increase was detected in patients affected by MS during clinical relapse; this finding was associated with a decrease in the number of CD19+/CD200+ (naïve B cells) EVs. Furthermore, an association emerged between gadolinium-enhanced MRI lesions in the CNS and the increase in the number of CCR3/CCR5 (subset of CD8 memory T cells), CD4/CCR3 (Th2 cells), and CD4/CCR5 (Th1 cells) CSF EVs, emphasizing again EVs as a pivotal and promising biomarker of inflammatory activity [48].

In another study, Masvekar and colleague did not find a difference between levels of EVs deriving from cell apoptosis (apoptosomes) isolated in CSF of RRMS (active and non-active) patients and HC, suggesting that apoptotic bodies are not appropriate as disease biomarkers [65], but further validations are necessary.

### 3.3. EVs as a Biomarker of Pharmacological Response

EVs have been proposed as an accessible predictive marker of the pharmacological response to disease-modifying therapies (DMT) in MS. Several studies contributed in this sense to expand current knowledge. Preclinical and clinical studies based on treatment with the second line oral drug fingolimod (FGM), the founder of sphingosine 1-phosphate receptor modulator drugs, showed a considerable reduction in microglia-derived EVs in the CSF of both active and non-active RRMS and PPMS patients and in EAE mice [16]. Such an effect was expected considering that FGM is a specific inhibitor of sphingomyelinase acid potentially involved in EV release. In the long term, a changing of the EVs content was also observed, consisting in a differential expression of miRNA and other compounds implicated in recovery from damage. In another study conducted on a court of active and non-active RRMS patients under FGM treatment, Zinger and colleagues observed that endothelial EVs (CD 105+) were significantly higher in MS patients relatively to HC and that FGM treatment was able to reduce their levels, reaching values similar to HC. In support of these results, the authors showed a reduced formation of surface blebs in human brain endothelial cells pre-cultured for 24 h with FGM and exposed to TNF-α for 18 h [66]. Curiously, the author observed that EVs derived from B cells (CD19+) were less in MS untreated patients compared to HC and that were significantly increased after FGM administration [66]. This differential effect of FGM on EVs release was not investigated. Similarly, in a study conducted by Sáenz-Cuesta and colleagues, FGM treatment induced an early enhancement of the EVs content in the plasma, mainly represented by platelet-derived (CD61+), leukocyte-derived (CD45+), and monocyte-derived (CD14+) EVs. The author also showed that the inhibitory role on lymphocyte activation exerted by circulating EVs was reduced following FGM treatment and observed a modulation of the EV miRNA cargo (miRNA not specified) [49]. Of note, such effects were observed after 5 h from the treatment and were interpreted by the authors as a consequence of the microenvironmental changing induced by FGM; lymphocyte arrest in lymph nodes induced by FGM may result in a low inflammatory status at peripheral level, where more EVs with a low regulatory profile are released. Based on all this evidence, FGM treatment certainly exerts different effects on EV depending on the cell-type origin, MS status, and duration of the treatment. Multiple mechanisms and concomitant factors might contribute to providing different effects on EVs, making difficult to compare results among these diverse studies. Furthermore, the lack of a deep knowledge of the EV function and of standardization in the methodology does no help in clarifying this complex scenario.

Similarly, it has been observed that IFN-β, a first-line treatment for RRMS patients, was able to stabilize the injured endothelium in association with a decrease in CD31+/PECAM-1+, CD54+, and CD146/intercellular adhesion molecule (ICAM-1) + EVs [62]. Conversely, in a different work, RRMS patients treated with IFN-β or natalizumab had significant higher counts of three EV subtypes (platelets-, total leukocytes-, or monocytes-derived) compared with untreated patients [45]. The mechanisms underlying such effects were not fully elucidated. Finally, a recent study conducted on a population of non-active RRMS patients treated with IFN-β or drug naïve showed a different exosomal miRNA profiling between RRMS treated and untreated patients, suggesting exosomal miRNA cargo as a possible prognostic tool of drug response [56].

Altogether, these data clearly highlight the relevance of EVs as potential biomarkers for diagnosis and therapeutic response in MS, however validated and reproducible results are still missing. Future research toward the screening of specific EVs subsets based on their cargo and membrane compositions associated to specific MS pathogenetic mechanisms might help guiding MS diagnosis, prognosis, and response to therapy. In the following paragraphs, we gained insight into the involvement of EVs in specific MS pathogenic mechanisms.

## 4. EV-Mediated Blood-Brain Barrier Dysfunction and Peripheral Immune Response

**Figure 1:**
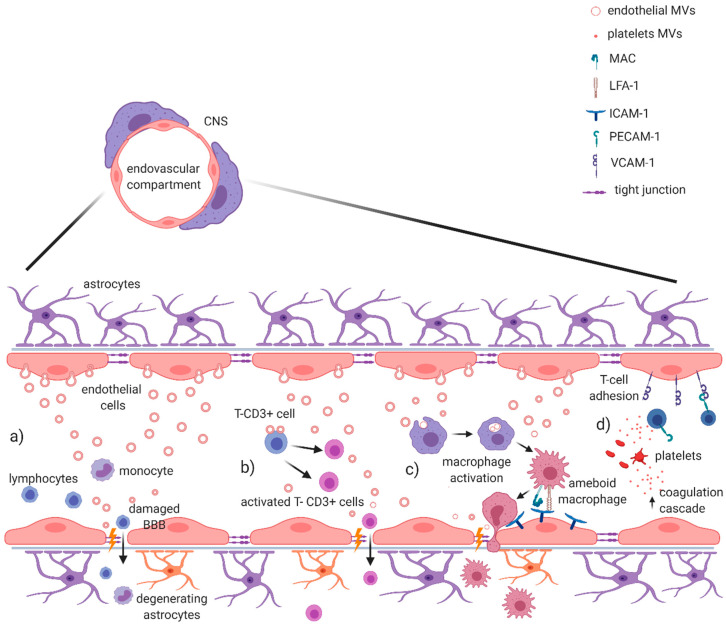
EV-mediated blood–brain barrier dysfunction in MS and EAE. It has been demonstrated that EVs are capable of damaging the integrity of blood–brain barrier during active phase of MS. (**a**) In the endovascular compartment, endothelial EV production directly promotes the disruption of extracellular matrix and tight junctions, allowing the passage of leukocytes through BBB. (**b**) Endothelial EVs are also directly involved in the activation of T-CD3+ cells, contributing to the BBB damage. (**c**) Other evidence suggests a role in monocyte/macrophage activation, with the expression of Mac and LFA-1 integrins that promote transendothelial migration of activated macrophages. (**d**) Platelet-derived MVs contribute to enrich local inflammatory milieu, with an activation of coagulation cascade mediated by thrombin; at the same time, they help lymphocyte adhesion on endothelium binding CD31/PECAM-1 and increase the expression of VCAM-1, further contributing to the BBB damage. Figure created with BioRender.com.

In MS, during BBB dysfunction, a pro-inflammatory microenvironment induces the endothelium to release cytokines and metalloproteinases also through endothelial EVs, promoting disruption of the extracellular matrix and endothelial tight junctions (Figure 1a). This process increases paracellular leakage of soluble mediators and activates leucocytes into the CNS [45,67,68]. In clinical studies, it has been demonstrated that endothelial EVs (Annexin V+, CD31/PECAM-1+) obtained from serum of HC were involved in the activation of CD4+ and CD8+ T-lymphocytes through expression of b2-microglobulin, MHC II, CD40, and inducible T cell costimulator ligand (ICOSL) [43], while endothelial EVs, isolated from the serum of active RRMS patients, were able to also promote the trans-endothelial migration of monocytes through a monolayer model of BBB. These activated monocytes express Mac-1 and LFA-1 integrins that are receptors for endothelial ICAM-1 [41,44,45]. Of note, EVs shaded from T-cells can enhance the expression of Mac-1, on the surface of monocytes, and ICAM-1, on endothelial cells. This effect was associated to chemokine CCL5 and arachidonic acid contained in T-cells EV [52,69] (Figure 1b,c).

Pro-inflammatory microenvironment has been also associated to EVs shedding from platelets (glycoprotein Ib alpha chain/CD42b+), significantly increased in plasma of MS patients [42,47,70]. Marcos-Ramiro and colleagues isolated platelet EVs and endothelial EVs from the serum of CIS, RRMS and PMS patients and verified their effects on the permeability of a monolayer model of BBB by using trans-endothelial electric resistance (TEER; a measure inversely proportional with barrier permeability) and by confocal analysis. PMS endothelial EVs were able to reduce TEER, causing discontinuity of the experimental barrier, as confirmed also by confocal analysis. Notably, although RRMS endothelial EVs were associated to experimental barrier disruption only in the confocal analysis, reduced TEER occurred in the co-presence of platelet EVs. Finally, CIS endothelial EVs and platelet EVs were not able to influence experimental barrier permeability. Those data seem to support the growing relevance of platelet and platelet EVs in MS. In particular, platelet EVs containing thrombin seems to amplify and modify inflammation at the level of the endothelium, since thrombin acts as an important constituent of innate immunity cross-talking coagulation cascade [71] (Figure 1d). Accordingly, platelet depletion or thrombin inhibition ameliorated neurological symptoms of EAE mice [47]. Platelet EVs are also capable to bind CD31/PECAM-1 on lymphocytes, increasing expression of VCAM1 integrin and promoting the adhesion of these cells to the endothelium [45,46] (Figure 1d). This is an important process in the pathogenesis of MS, considering that inhibition of T cells entry into the CNS underlines the mechanism of action of Natalizumab, a humanized monoclonal antibody against very late antigen (VLA)-4 currently used to treat RRMS [72]. Altogether, this evidence suggests that MS EVs potentiate the transmigration of lymphocytes and myeloid cells through the BBB and thus facilitate this critical pathogenic step in the development of disease in MS.

An interesting association between plasmatic EVs and T lymphocyte activation in the EAE model has been also reported [73] (Figure 1). Administration of EVs derived from plasma of C57BL/6 mice into mice with active EAE induced a distinct spontaneous relapsing−remitting EAE phenotype characterized by a prominent contribution of CD8+ T cells, while EAE is primarily mediated by a prominent CD4+ lymphocyte activation [73]. The authors showed that fibrinogen in plasmatic EVs cargo was required to induce a spontaneous relapsing−remitting activity in EAE mice, providing evidence that this type of EVs can influence autoimmune responses in a model of MS. The analysis of plasmatic EVs from RRMS patients identified fibrinogen as portion of the cargo with no apparent differences in comparison to control EVs samples. The authors suggested that the presence of fibrinogen-plasmatic EVs per se was not responsible for such effect, likely derived by a synergic interaction with inflammatory milieu.

Finally, an impaired function of regulatory T cells (Tregs) has been linked to the pathogenesis of MS. Interestingly, it has been shown that exosomes derived from Treg of RRMS patients were less effective in suppressing proliferation of conventional T cells (Tconv) and in inducing Tconv apoptosis in comparison to exosomes derived from HC [55] (Figure 1). On the other hand, Tregs can be modulated by EVs action. Kimura and colleagues demonstrated by in vitro experiments that exosomes circulating in MS patients were able to reduce the relative frequency of IFN-γ−IL-17A−Foxp3+CD4+ T cells, regarded as most suppressive Treg cells. In particular, the authors showed that miR-Let-7i was increased in MS exosomes and was responsible of the inhibition of Treg cells differentiation from naive CD4+ T cells [54]. On the other hand, a protective role for miR-Let7 recently emerged in the EAE model, being involved in the inhibition of T helper-17 differentiation, which is a crucial step for EAE development [74]. Further investigations are therefore necessary to clarify its role in MS pathophysiology.

## 5. EVs Involvement in CNS Inflammatory Processes

**Figure 2:**
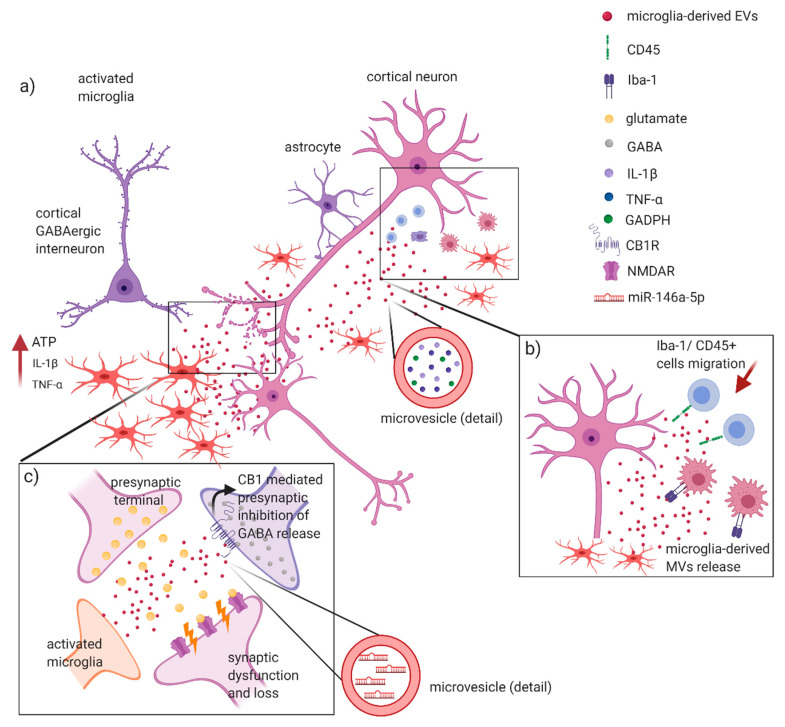
Glia-derived EVs involvement in MS and EAE inflammatory processes. Glia cells play a central role in triggering and sustaining inflammatory processes in MS/EAE. It has been suggested that: (**a**) Release of ATP from damaged cells, in association with IL-1β and TNF-α, leads to the activation of microglia with secretion of MVs containing proinflammatory cytokines, such as IL-1β and TNF-α, and glycolytic enzymes (GADPH). These molecules favorite the spreading of the inflammatory stimulus in the CNS; (**b**) microglia-derived MVs are capable of recruiting Iba-1 and T-CD45+ cells, with the generation of inflammatory foci in CNS underlying demyelination and axonal loss in EAE; (**c**) these events can potentially lead to synaptic dysfunction, causing an enhanced release of glutamate in the synaptic cleft with an aberrant activation of NMDA receptors. At the same time, microglia-derived MVs might activate presynaptic cannabinoid receptors type 1 (CB1R), thus inhibiting release of GABA by cortical interneurons. Furthermore, miR-146a-5p upregulated in EVs secreted from reactive microglia may induce synaptic loss and dysfunction in the recipient neurons. Figure created with BioRender.com.

Several studies recently suggested the involvement of glial EVs in the proinflammatory processes of the CNS [52,75]. Microglia, the major players of neuroinflammation, communicate with the other CNS cells by secreted vesicles in both physiological and pathological conditions [10]. It has been shown that microglial EVs and particularly MVs are enriched in pro-inflammatory mediators, such as IL-1β [67] and TNF-α [76], and the glycolytic enzyme glyceraldehyde 3-phosphate dehydrogenase (GAPDH) [77], thus propagating inflammatory stimulus throughout the brain and enforcing inflammation in neuroinflammatory diseases such as MS [78] (Figure 2a). As already mentioned, MS and EAE are characterized by the rapid recruitment of blood-borne monocytes, the reaction of resident microglia and perivascular macrophages, along with the recruitment of T cells [20]. Activated microglia and macrophages can be found in white matter lesions (early and late) and in gray matter subpial lesions [79]. Macrophages within CNS lesion sites are difficult to distinguish from reactive microglia, since they both are amoeboid-shaped and express the same antigenic markers. Clinical evidence reported an increase in microglia/macrophage MVs in the CSF of both CIS and relapsing RRMS patients, correlating with a predominance of inflammatory processes. Accordingly, preclinical studies provided evidence that injection of MVs derived from cultured microglia into the corpus callosum of EAE mice promote infiltration of CD45+ cells and high recruitment of ameboid ionized calcium-binding adapter molecule 1 (Iba1)+ cells close to the site of injection, suggesting a microglia MV’s involvement in the formation of inflammatory foci in the EAE environment [16] (Figure 2b). Moreover, an increase in reactive microglia/macrophage MVs detected in the CSF of EAE mice has been associated to a worse course and major severity of disease [16]. Furthermore, the authors showed that knock-out mice of Acid SMase (A-SMase KO mice), a specific enzyme of sphingomyelinases family necessary for EVs shedding from glial cells [80], were highly resistant to the development of EAE, confirming that MVs are fundamental in sustaining EAE pathology [16]. However, other studies reported that inhibition of A-SMase impairs IL-6 production [81] and reduce T cell transmigration across the BBB [82], suggesting that A-SMase covers more than one key point role in MS pathophysiology [83]. The impact of microglia/macrophages EVs on EAE disease has been further supported by an elegant study based on the use of engineered BV2 cells in order to release EVs targeting phagocytes and containing the anti-inflammatory cytokine IL-4 [84]. A single injection of these exogenous EVs into the cisterna magna of EAE mice at the onset of the disease resulted in an improvement of clinical score and reduction in inflammatory infiltrates, demyelination, and axonal loss in the spinal cord of these mice [83].

Altogether these finding propose microglia EVs as important mediators of inflammation, contributing at propagating neuroinflammatory stimuli in the CNS. Of note, emerging evidence implicates microglia also as active players in neuronal network formation and information processing, by regulating synapse number and modulating synaptic function and plasticity (Figure 2c). Remarkably, microglia-derived MVs appear to regulate the excitatory–inhibitory balance of neurotransmission through several independent mechanisms. Microglia-derived EV, for instance, critically enhanced glutamatergic transmission [85], while they were able to activate presynaptic cannabinoid receptor type 1 (CB1) expressed by GABA-ergic neurons, inhibiting thus presynaptic transmission [86]. Interestingly, in a recent study emerged a role for miR-146a-5p upregulated in EVs secreted from reactive microglia either exposed to inflammatory or degenerative stimuli [57]. Upon EV fusion with the plasma membrane, miR-146a-5p is delivered to neurons where represses translation of presynaptic synaptotagmin-1 and postsynaptic neuroligin-1 with an impact on synaptic density and transmission (Figure 2). Notably, the same authors validated the presence of miR-146a-5p in the EVs isolated from the CSF of MS patients [57].

A bidirectional interaction between neuron and microglia mediated by EVs has been also proposed. It has been indeed shown that exosomes released by neurons promote microglial pruning of degenerating neurites, thus suggesting a role for exosomes as regulators of synapse elimination [87].

Studies on the role of microglial EVs in MS inflammatory synaptopathy [88] are lacking, but the observation that EVs can participate in synaptic dysfunction and loss through several mechanisms (inflammatory and non-inflammatory) is an intriguing aspect that deserves future investigations.

Finally, microglia participate in both myelin injury and remyelination during MS [30]. The involvement of microglia EVs in these processes is described in the next paragraph.

## 6. Potential Involvement of EVs in Remyelinating Processes

**Figure 3:**
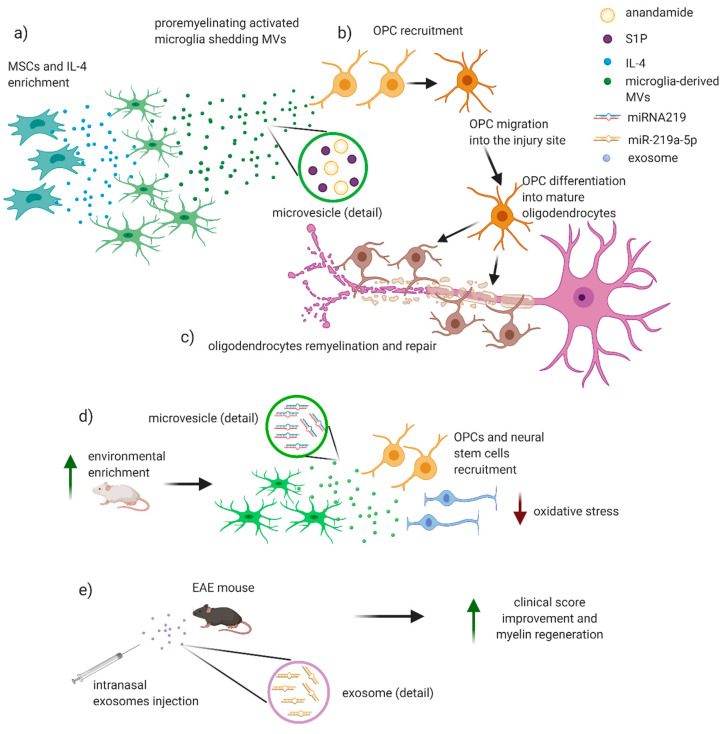
Potential role of EVs in remyelinating processes in MS/EAE. EVs may have a potential role in remyelination and reparative processes in MS/EAE. (**a**) Incubation of microglial cells with IL-4 and MSCs allows the developing of a pro-regenerative microglial phenotype that secretes MVs containing anandamide and sphingosine 1 phosphate (S1P). (**b**) These molecules are strong chemoattractants for oligodendroglial precursor cells (OPCs) that migrate near the injury site and differentiate into mature oligodendrocytes, (**c**) wrapping on damaged axons and thus restoring the integrity of myelin sheet. (**d**,**e**) Recent works put into evidence an emerging role for microRNA 219 (miR-219) in remyelination. (**f**) Exposition to an incremented physical, intellectual, and social activity in rats leads to an anti-inflammatory microglia with release of exosomes containing miRNA 219 that is associated to an increase in myelin content, OPCs, and neural stem cells, with a consequent reduction in levels of oxidative stress. (**g**) Intranasal injection of exosomes enriched with miR-219a-5p significantly improved the clinical score of EAE mice, likely promoting myelin regeneration. Figure created with BioRender.com.

Direct evidence of the involvement of EVs in demyelinating/remyelinating processes in EAE/MS are still lacking. However, since 1989, oligodendroglial MVs in the CSF of patients with MS have been associated to oligodendrocytes activation and myelin injury repair [64] (Figure 3). In a recent work, it has been investigated the effects of EVs, produced in vitro by either pro-inflammatory or pro-regenerative microglia, on OPCs at demyelinated lesions caused by lysolecithin injection in the mouse corpus callosum. Interestingly, EVs released by pro-inflammatory microglia blocked remyelination, whereas EVs produced by microglia co-cultured with immunosuppressive MSC promoted OPC recruitment and myelin repair (Figure 3). By using primary OPC cultures, the authors dissected the molecular mechanisms responsible for the harmful and beneficial EVs actions. OPCs cultured either alone or with astrocytes were exposed to inflammatory EVs. A blockade of OPC maturation was observed only in the presence of astrocytes, implicating these cells in remyelination failure. The authors suggested that astrocytes may be converted into harmful cells by the inflammatory EVs cargo whereas surface lipid components of EVs promote OPC migration and/or differentiation, linking EVs lipids to myelin repair. The authors highlighted that pro-regenerative EVs, obtained by incubation of activated microglia with IL-4 and MSCs, contained high levels of anandamide and sphingosine 1 phosphate (S1P), two lipids that together with the protein Wnt3a share a key chemoattractant function for OPCs [36] (Figure 3a,b). The relevance of S1P derived also by the evidence of a beneficial effect on remyelination mediated by S1PR modulators, a group of drugs used in the MS therapy [36,89] (Figure 3c). It emerged also the role of A1-astrocytes in mediating the anti-remyelinating EVs effects on OPCs. Of note, such phenotype is obtained when astrocytes are exposed to IL-1α, TNF-α, and C1q released by activated microglia, and it has been recently proven to slow OPC differentiation and to damage differentiated oligodendrocytes. Of note, this specific phenotype has been observed in demyelinating plaques of MS patients [36,90]. Similarly, after stimulation with low-level IFN-γ, dendritic cells (DCs) can release exosomes that increase remyelination following acute lysolecithin-induced demyelination [91]. Other authors suggested that pro-remyelinating effect of EVs is also mediated by miRNA-219 (Figure 3d,e). This miRNA is increased in the serum exosomes, released by pro-remyelinating microglia, crossing the BBB of rats exposed to environmental enrichment (increased physical, intellectual, and social activity) (Figure 3f). The effect of miRNA-219 was previously associated to an increase in myelin content and amount of OPC and neural stem cells [92]. Together with miRNA-219, S1P directly drives maturation of OPCs into mature oligodendrocytes. Interestingly, it has been observed that intranasal injection of exosomes enriched with miR-219a-5p significantly improved the clinical score of EAE mice, likely promoting myelin regeneration [58] (Figure 3g). Furthermore, it is reasonable that EVs protein cargo may indirectly regulate OPCs maturation by influencing the microenvironment around the lesion in a way that has still to be clarified [36].

Finally, in physiological conditions oligodendrocytes also provide trophic support to neurons, a process critical for long-term axonal integrity, by releasing exosomes containing heat shock proteins and antioxidants, together with mRNA and miRNAs. Increased levels of cytoplasmic Ca2+, mediated by activation of both oligodendroglial N-Methyl- d-aspartate (NMDA)-type and, to a lesser extent, α-amino-3-hydroxy-5-methyl-4-isoxazolepropionic acid (AMPA)-type glutamate receptors, trigger exosome release from oligodendrocytes. Those exosomes concentrate into the periaxonal space and are endocytosed by neurons at axonal and somatodendritic sites [78]. A dysregulation of such exosome-oligodendrocyte trophic support to axon is plausible in MS disease but has not yet investigated and remains a point of interest for future research.

Altogether, these observations reinforce the importance of providing a deeper characterization of the function, cargo, and origin of EVs in both physiological and pathological conditions. Particular attention should be given to the distinct astrocytic and microglia phenotypes (beneficial or detrimental) that may coexist or develop sequentially during different phases of a pathological process. Their identification through isolation and characterization of circulating EVs might be really helpful to early define ongoing pathological processes and potentially provide targeted therapeutic interventions.

## 7. New Therapeutic Perspectives

Based on the fact that EVs allow intercellular communication and can modulate the phenotype of target cells, EVs can be regarded as potential therapeutic targets and tools for therapeutic intervention. Accordingly, many preclinical studies reported that inhibition of MVs release or selective blockade of MVs components can reduce local propagation of several neurodegenerative and neuroinflammatory diseases [93,94]. At the same time, their good biocompatibility, high targeting specificity, and low immunogenicity have suggested EVs as carriers of small therapeutic molecules, proteins and nucleic acids targeting CNS in neurodegenerative disorders [22,23]. The generation of engineered microvesicles with a marked organotropism towards the target tissues has allowed the development of nano-based drug delivery system technologies, laying the foundations for an extremely promising branch of personalized medicine. Alvarez-Erviti and colleagues demonstrated that engineered EVs containing small interfering RNA (siRNA) and injected in blood vessels could pass the BBB and interfere with genes involved in the synthesis of amyloid precursor protein in a mouse model of Alzheimer’s disease [95]. Other translational studies subsequently confirmed the effectiveness of MVs in being delivery cargoes for immunological therapies in brain cancers [96] and, more generally, for drug delivery and vaccination [97]. Clinical application of modified EVs derived from MSCs has been successful in ischemic stroke, showing a recovery from brain injury in both the acute and chronic phases and the release of paracrine factors that promoted brain plasticity [98]. MSCs represent an attractive alternative to develop a cell-based therapy for MS. MSCs display stromal features and exert bystander immunomodulatory and neuroprotective activities. MSC have been employed into recovery from myelin damage both in clinical and preclinical studies, and there is evidence that EVs from MSC contribute to exert a beneficial effect [99,100]. Of note, therapeutic applications of EVs have been developed also for MS especially as far as concern tissue repair in preclinical studies. Exosomes produced by human MSC stimulated with IFNγ and administrated intravenously to EAE mice could induce remyelination, ameliorate clinical score, and dampen neuroinflammation. These beneficial effects were associated to an increased number of CD4+CD25+FOXP3+ Tregs within the spinal cords, a reduced release of pro-inflammatory cytokines (including IL-6, IL-12p70, IL-17AF, and IL-22), suggesting exosome-MSCs as a promising therapeutic tool for MS patients [101]. Similarly, Li and colleagues showed the beneficial effect of MSC-exosome in rat EAE model by addressing their modulation on microglia polarization. Engineered EVs emerged also as a great potential nano-therapeutic tool. MSC exosomes armed with high affinity aptamer toward myelin were administrated to EAE model and produced a robust suppression of inflammatory response as well as lowered demyelination lesion region in CNS, resulting in reduced severity of EAE disease [102]. Mokarizadeh and colleagues addressed in vitro the potentiality of exosomal nano-shuttles as a novel approach for phenotype modification of auto-reactive cells, when incubated with EAE mice splenic lymphocytes. MSC-derived exosomes expressing TGF-β, PD-L1, and Gal-1 were able to induce inhibition of auto-reactive lymphocyte activation and proliferation by transferring these tolerogenic molecules. Such effect was also due to promotion of CD4+ CD25+ Foxp3+ Treg generation and apoptotic activity towards activated T cells [103]. Besides MSC-derived exosomes, the therapeutic potential of MVs from other cell types has been investigated. In a recent elegant study with the aim of targeting phagocytes, BV2 microglia cells were engineered to release MVs containing IL-4 and to overexpress the endogenous “eat me” signal Lactadherin (Mfg-e8) on their surface. A single-dose injection of these EVs in the cisterna magna of EAE mice induced a reduction in clinical symptoms, promoting tissue repair through a STAT-6-dependent anti-inflammatory activity [84]. The great potential for use of EVs as a therapeutic to promote remyelination in MS has been also supported by experiments conducted with exosomes released by DC cultures stimulated with low-level IFNγ (IFNγ-DC-Exos). These IFNγ-DC-exosomes contained microRNA that could increase baseline myelination, reduce oxidative stress, and improve remyelination following acute lysolecithin-induced demyelination [91].

## 8. Conclusions

In conclusion, the present review provides an update on EVs in MS and its mouse model EAE. We first reported clinical studies showing associations of MVs circulating in MS biological fluids (CSF and plasma) with clinical parameters, activation of cells contributing to MS pathogenesis, and with therapeutic response. Several authors have proposed EVs as plausible biomarkers especially related to the active phase of the disease. However, the heterogeneity of MS disease as well as the lack of a proper standardization of MVs measurements has made difficult to translate into clinical practice the use of MS as reliable biomarkers. To overcome this issue, researchers might improve the EVs isolation methodology, an important step to obtain a valid characterization of their function. Choosing the right isolation method is indeed critical; the nature of biofluids, the mechanism of isolation, and the throughput of the method should be carefully evaluated and developed to allow the routine detection in individual samples. Proteomic, transcriptomic as well as morphological analyses are also crucial steps to deeply characterize EVs. Furthermore, it would be important to use valid experimental models to reveal functions strictly linked to a specific MS pathogenic mechanism. The application of chimeric ex vivo MS models (CSF MS or T-cell MS chimeric models, [39,104,105]), consisting in incubation of EVs derived from MS biofluids onto mouse brain slices, together with biochemical, molecular, and electrophysiological analysis, might represent a good tool to study EVs roles in MS pathology.

Regardless of the methodology, we analyzed the current literature specifically related to the EVs involvement in each pathogenic mechanism underlying MS/EAE disease. Accumulating evidence from clinical and prevalently from preclinical studies convincingly showed that MVs released from BBB-endothelial cells, platelets, leukocytes, myeloid cells (monocytes/macrophages/microglia), astrocytes, and oligodendrocytes are involved in the pathogenesis of MS/EAE. Most of the information points to an impact of EVs on BBB damage, on spreading pro-inflammatory signals, and altering neuronal functions. On the other hand, an attractive reparative functions of EVs emerged from the complex interaction between neurons, oligodendrocytes, microglia, and astroglia cells. Notably, demyelination and synaptopathy are potentially reversible phenomena in MS disease that deserve attention as potential targets for EVs-based therapeutic strategies. In this regard, EVs derived from MSCs and immune cells can potentiate tissue regeneration, take part in immune modulation, and function as potential alternatives to stem cell therapy, and bioengineered EVs can act as delivery shuttle for therapeutic molecules.

## Figures and Tables

**Table 1:** Extracellular vesicle (EV) classification and their potential role in multiple sclerosis (MS).

EVs Cellular Origin	Surface Marker	Functional Implication	Detection Levels	References	Study Size
**Serum/Plasma**					
**Endothelial cells**	CD31+	Acute BBB disruption and contribution in Gad+ MRI active lesions	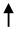 E-MS 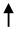 RRMS 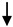 R-MS; SPMS; HC	Minagar et al., 2001 [40]	48 HC; 30 E-RRMS; 20 R-RRMS
				Jy et al., 2004 [41]	35 HC; 30 E-RRMS; 20 R-RRMS
				Alexander et al., 2015 [42]	36 HC; 44 RRMS; 16 SPMS
		CD4+ and CD8+ T-lymphocytes activation		Wheway et al., 2014 [43]	
	CD51+	Chronic endothelial injury	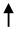 E-MS; R-MS 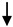 HC	Minagar et al., 2001 [40]	48 HC; 30 E-RRMS; 20 R-RRMS
	CD54+ CD62E CD106+	Monocytes conjugates for endothelial adhesion	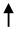 E-MS 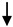 R-MS; HC	Jy et al., 2004 [41]	35 HC; 30 E-RRMS; 20 R-RRMS
				Jimenez et al., 2005 [44]	10 HC; 11 E-RRMS; 9 R-RRMS
**Monocytes**	CD14+	Acute endothelial injury	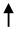 R-RRMS 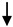 HC and SPMS	Saenz-Cuesta et al., 2014 [45]	20 HC; 13 SPMS 64 R-RRMS
**Leukocytes**	CD45+	Acute endothelial injury	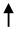 R-RRMS 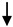 HC and SPMS	Saenz-Cuesta et al., 2014 [45]	20 HC; 13 SPMS 64 R-RRMS
**Platelets**	CD62p CD41+/ CD61+	Platelets activation and leukocytes interaction with damaged endothelium	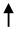 R-MS (RRMS) 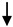 HC and SPMS	Saenz-Cuesta et al., 2014 [45]	20 HC; 13 SPMS 64 R-RRMS
				Sheremata et al., 2008 [46]	92 HC; 33 R-RRMS
	CD42b+	Incremented experimental BBB permeability (except for CIS)	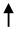 MS (PMS; RMS; CIS) 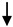 HC	Marcos-Ramiro et al., 2014 [47]	49 HC; 23 SPMS; 51 RRMS; 12 CIS; 9 PPMS
		**Cerebrospinal Fluid**			
**Microglia/Macrophage**	IB-4	Acute BBB disruption and contribution in Gad+ MRI active lesions	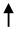 E-MS (CIS, RRMS) 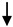 R-MS (RRMS); HC	Verderio et al., 2012 [16]	13 HC; 39 R- RRMS 28 E-RRMS; 28 CIS
**T-cells**	CCR3/ CCR5 CD4/ CCR3 CD4/ CCR5	Acute BBB disruption and contribution in Gad+ MRI active lesions	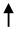 E-MS (RRMS) 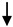 R-MS (RRMS)	Geraci et al., 2018 [48]	10 R-RRMS; 13 E-RRMS

Abbreviations: BBB (blood–brain barrier); E-MS (exacerbated-MS); R-MS (remission-MS); HC (healthy controls); RRMS (relapsing–remitting MS); SPMS (secondary progressive MS); CIS (clinically isolated syndrome); OPC (oligodendroglial precursor cells). Up and down arrows refer to high and low levels of EVs, respectively.

**Table 2:** Classification of miRNA content in EVs involved in MS.

miRNA	Detection Level	Study Size	Functional Implication	Reference
**miR-15b-5p**	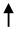 In RRMS vs. HC	14 RRMS 11 S/PPMS 11 HC	Targets FGF-2 implicated in demyelination and remyelination	Ebrahimkhani et al., 2017 [51]
**miR-451a**	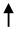 In RRMS vs. HC	14 RRMS 11 S/PPMS 11 HC	Regulator of oxidative stress	Ebrahimkhani et al., 2017 [51]
**miR-30b-5p**	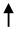 In RRMS vs. HC	14 RRMS 11 S/PPMS 11 HC	Neuro-axonal injury	Ebrahimkhani et al., 2017 [51]
**miR-342-3p**	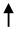 In RRMS vs. HC	14 RRMS 11 S/PPMS 11 HC	Neuro-axonal injury	Ebrahimkhani et al., 2017 [51]
**miR-127-3p**	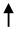 In S/PPMS vs. HC	14 RRMS 11 S/PPMS 11 HC		Ebrahimkhani et al., 2017 [51]
**miR-370-3p**	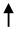 In S/PPMS vs. HC	14 RRMS 11 S/PPMS 11 HC		Ebrahimkhani et al., 2017 [51]
**miR-409-3p**	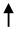 In S/PPMS vs. HC	14 RRMS 11 S/PPMS 11 HC		Ebrahimkhani et al., 2017 [51]
**miR-432-5p**	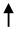 In S/PPMS vs. HC	14 RRMS 11 S/PPMS 11 HC		Ebrahimkhani et al., 2017 [51]
**miR-122-5p**	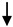 In remission RRMS vs. HC; 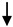 in relapse RRMS vs. remission RRMS	30 Remission- RRMS 33 Relapse- RRMS 32 HC	Targets STAT3 and AHR (not validated), regulators of differentiation of Th17 and immunosuppressive T cells	Selmaj et al., 2017 [53]
**miR-196b-5p**	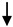 In relapse RRMS vs. HC; 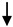 in relapse RRMS vs. remission RRMS	30 Remission- RRMS 33 Relapse- RRMS 32 HC	Targets STAT3 and AHR (not validated), regulators of differentiation of Th17 and immunosuppressive T cells	Selmaj et al., 2017 [53]
**miR-301a-3p**	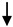 In relapse RRMS vs. HC	30 Remission- RRMS 33 Relapse- RRMS 32 HC	Targets STAT3 and AHR (not validated), regulators of differentiation of Th17 and immunosuppressive T cells	Selmaj et al., 2017 [53]
**miR-532-5p**	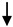 In relapse RRMS vs. HC; 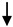 in relapse RRMS vs. remission RRMS	30 Remission- RRMS 33 Relapse- RRMS 32 HC	Targets STAT3 and AHR (not validated), regulators of differentiation of Th17 and immunosuppressive T cells	Selmaj et al., 2017 [53]
**Let-7i**	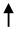 In MS vs. HC	4 MS 4 HC	Inhibition of Treg cells differentiation from naive CD4+ T cells	Kimura et al., 2018 [54]
**miR-146a-5p**	Detected in CSF exosomes	MS = 10	Synaptic alterations in in vitro experiments	Prada et al., 2018 [57]
**miR-219a-5p**	Artificially enriched exosomes	EAE mice	Maturation of OPCs; clinical score improvement	Osorio-Querejeta et al., 2020 [58]

MiRNA detected in EVs derived from serum and plasma of MS patients. Abbreviations: HC (healthy controls); RRMS (relapsing–remitting MS); SPMS (secondary progressive MS); PPMS (primary progressive MS); FGF-2 (fibroblast growth factor-2); STAT3 (signal transducer and activator of transcription 3); AHR (aryl hydrocarbon receptor); EAE (experimental autoimmune encephalomyelitis); OPCs (oligodendrocyte precursor cells). Up and down arrows refer to high and low levels of miRNA, respectively.

## Abbreviations

EVs	extracellular vesicles
MS	multiple sclerosis
RNAs	ribonucleic acids
CD	cluster of differentiation
mRNAs	messenger RNA
ncRNAs	non-coding RNAs
miRNAs	micro-RNA
MVEs	multivesicular endosomes
ESCRT	endosomal sorting complex required for transport
SMase	sphingomyelinase
MVs	microvesicles
T regs	regulatory T cells
EAAT2	excitatory amino acid transporter 2
GLT1CNS	glutamate transporter-1 central nervous system
RRMS	relapsing remitting MS
SPMS	secondary progressive multiple sclerosis
PPMS	primary progressive multiple sclerosis
CIS	clinically isolated syndrome
RIS	radiologically isolated syndrome
MOG	myelin oligodendrocyte glycoprotein
EAE	experimental autoimmune encephalomyelitis
BBB	blood–brain barrier
INF-γ	interferon-γ
IL-1β	interleukin 1β
TNF-α	tumor necrosis factor-α
VCAM	vascular cell adhesion molecule
PECAM	platelet endothelial cell adhesion molecule
MRI	magnetic resonance imaging
OPCs	oligodendroglial precursor cells
NG2	neural/glial antigen 2
OLGs	oligodendrocytes
MSC	mesenchymal stem cells
iNOS	inducible nitric oxide synthase
TMS	transcranial magnetic stimulation
GABA	gamma-aminobutyric acid
HC	healthy control
Gad+	gadolinium positive
PBMC	peripheral blood mononuclear cell
FGM	fingolimod
TEER	trans-endothelial electric resistance
VLA	very late antigen
Tconv	conventional T Cells
GAPDH	glyceraldehyde 3-phosphate dehydrogenase
Iba-1	ionized calcium-binding adapter molecule 1
A-SMase	acid sphingo-myelinase
CB1	cannabinoid receptor type 1
S1P	sphingosine 1 phosphate
DCs	dendritic cells
NMDA	N-methyl-d-aspartate
AMPA	α-amino-3-hydroxy-5-methyl-4-isoxazolepropionic acid
siRNA	small interfering RNA
ICAM	intercellular adhesion molecule
ICOSL	inducible T cell costimulator ligand
PMS	progressive multiple sclerosis
